# Loss of regional theta differentiation in TMS-EEG response marks network dysfunction in psychosis risk

**DOI:** 10.1038/s41398-026-04030-5

**Published:** 2026-04-10

**Authors:** Nadja Zimmermann, Matthias Liebrand, Chantal Michel, Miriam Stüble, Arndt-Lukas Klaassen, Eva Burkhardt, Roland Wiest, Michael Kaess, Jochen Kindler, Yosuke Morishima

**Affiliations:** 1 Translational Research Center, University Hospital of Psychiatry and Psychotherapy, University of Bern, Bern, Switzerland; 2 University Hospital of Child and Adolescent Psychiatry and Psychotherapy, University of Bern, Bern, Switzerland; 3 Graduate School for Health Sciences, University of Bern, Bern, Switzerland; 4 University Institute of Diagnostic and Interventional Neuroradiology, Inselspital Bern, Bern, Switzerland; 5 Child and Adolescent Psychiatry Baselland, Liestal, Switzerland

**Keywords:** Schizophrenia, Diagnostic markers

## Abstract

The clinical high-risk state for psychosis (CHR-P) aims to identify individuals in a potential prodromal phase of the disorder. Brain network dysconnectivity is believed to underlie psychotic disorders and has been studied using concurrent transcranial magnetic stimulation (TMS) and electroencephalography (EEG). However, such data is not yet available for individuals in the CHR-P state. Single-pulse TMS was applied to the left dorsolateral prefrontal cortex (lDLPFC), left posterior parietal cortex (lPPC), and dorsomedial prefrontal cortex (DMPFC) while measuring EEG. Time-frequency data in the gamma and theta bands were investigated in 58 healthy controls (HC) and 44 CHR-P individuals, testing for differences between stimulations sites and associations with psychopathology and neurocognition. We observed a differentiated response to TMS based on stimulation sites in the theta band in the HC group, with the strongest response being after lDLPFC stimulation, followed by lPPC and DMPFC. This differentiation was absent in CHR-P patients. The increased theta response after lPPC and DMPFC stimulation was negatively correlated with unusual thought content, while the response to lPPC and lDLPFC stimulation was negatively correlated with avolition, decreased experience of emotion, and deterioration in role functioning. Generally, no differences were found for gamma power. The absence of a differentiated theta response to TMS in CHR-P suggests the loss of functional specialization of the stimulated regions and their connections. Because of the inverse relationship between theta oscillations and psychopathological symptoms, we propose that this may represent a compensatory mechanism in the CHR-P state.

## Introduction

The clinical high-risk state for psychosis (CHR-P) was conceptualized to identify individuals who might develop a psychotic disorder as early as possible [1]. In addition to genetic risk factors accompanied by a decline in everyday functioning, the CHR-P state is characterized by early symptoms such as distorted perceptions and thinking, as well as subclinical psychotic symptoms [2]. About 25%–35% of people with CHR-P transition to full psychosis within four to ten years [3]. Current research focuses on refining early detection by combining psychopathological and biological factors to develop personalized risk assessments, tailored prevention, and novel treatment strategies [4, 5]. Thus, a better characterization of the neurophysiological basis for the pathophysiological roots of psychosis and its prodromal stages is pivotal.

The dysconnection hypothesis of psychosis suggests that a failure of functional integration in distributed neuronal systems may lead to aberrant – such as increased or decreased – interactions/connectivity among brain networks [6]. Possible neural structural and functional alterations underlying psychotic disorders have been widely studied with neuroimaging techniques such as Magnetic Resonance Imaging (MRI). Multiple distinct grey [7, 8] and white matter [9, 10] reductions as well as increased and decreased structural and functional connectivity [11] have been reported, often in relation to the frontoparietal network (FPN). Crucially, similar results have been found in first episode psychosis (FEP) and CHR-P individuals [12, 13]. In particular, dysconnections in frontal regions, such as subgenial corpus callosum and anterior corona radiata, and in the fronto-parietal network, the superior longitudinal fasciculus have been confirmed in recent mega-analysis studies [8–10, 12].

Neural oscillations are rhythmic or repetitive patterns of neural activity in the brain [14]. These oscillations are observed in electroencephalography (EEG) as a result of the synchronized activity of large populations of neurons and are thought to reflect the structural and functional organization of brain networks [15, 16]. Different frequency bands have been associated with distinct levels of cortical integration: fast gamma oscillations are believed to support local synchronization, whereas slower theta rhythms are implicated in long-range communication across brain regions [17]. In schizophrenia, a broad body of evidence points to aberrant oscillatory activity such as reduced gamma oscillations [18], suggesting that disrupted neural synchronization may be a core feature of the disorder. To better understand these disruptions, techniques that directly probe neural dynamics are essential.

Pairing TMS (transcranial magnetic stimulation) with EEG enables us to directly evoke activity in the stimulated brain region and track the spread of the activity due to the high temporal resolution of the EEG, allowing us to evaluate cortical reactivity and connectivity [19]. TMS-EEG thus offers a powerful, non-invasive approach for identifying biomarkers in psychiatric disorders like schizophrenia [20]. So far, TMS-EEG has not been applied to study the CHR-P state, but has revealed aberrant oscillatory activity in both chronic and first-episode psychosis patients. One consistent finding is the reduction in localized gamma power following the stimulation of the premotor cortex [21–23], motor cortex [23, 24], posterior parietal cortex, and prefrontal cortex [23, 25, 26].

Regarding lower frequency bands, frontal stimulation in patients has been associated with an early reduction in power, followed by prolonged activation in delta, theta, and alpha frequencies [27]. Motor cortex stimulation similarly leads to a reduction in delta and theta power [24]. One study [28] however points to another direction, with motor cortex stimulation producing no difference between healthy controls (HC) and schizophrenia patients in the initial EEG response. Recurrent excitation in response to TMS in patients was however observed in the gamma, theta, and delta bands, with gamma activity being correlated with positive and theta and delta with negative symptoms. In sum, the oscillatory response to TMS seems attenuated in patients, however, some studies also show prolonged or recurrent activity, which was also linked to symptom severity.

The aim of the current study was to investigate TMS-related oscillatory activity in CHR-P patients compared to HCs to evaluate how the assumed dysconnection is represented by aberrant oscillatory responses to TMS in the EEG. Reductions in gamma [21–26] and theta band activity [24, 27, 28] have been reported in psychotic samples, making these frequency bands of particular interest. As TMS targets, we chose the following three brain areas: the left dorsolateral prefrontal cortex (lDLPFC), dorsomedial prefrontal cortex (DMPFC), and left posterior parietal cortex (lPPC), based on previous research in structural dysconnections in prefrontal regions and FPN [8–10, 12].

We hypothesize that CHR-P individuals will show dysregulated – possibly reduced – TMS-related theta and gamma activity, reflecting early functional manifestations of dysconnection in FPN linked to emerging psychopathology and cognitive impairments.

## Methods and materials

### Participants

Forty-nine help-seeking individuals with a CHR-P state and nine help-seeking FEP patients were recruited from the Early Detection and Intervention Center (FETZ) Bern [29]. Fifty-nine HC were recruited from schools, universities, and the online recruitment tool of the University Hospital of Child and Adolescent Psychiatry and Psychotherapy in Bern (https://www.upd.ch/de/forschung/kinder-und-jugendpsychiatrie/studien.php). One HC and five CHR-P patients were excluded from analyses due to insufficient TMS-EEG data quality, resulting in *n* = 44 CHR-P and *n* = 58 HC. As the sample size for FEP was inadequate, this group was not included in analyses and are only reported in supplementary materials. The study was conducted in compliance with the Declaration of Helsinki [30] and approved by the Kantonale Ethikkommision (KEK) Bern (2021-00122) prior to the start. Participants provided their written informed consent. In accordance with the Human Research Act (HRA) [31] Art. 3k and Art. 23 in Switzerland, participants between the ages 14 to 18 were considered adolescents and signed the consent form themselves.

The presence of a CHR-P state was defined by meeting ultra-high-risk (UHR) [32] and/or basic symptom (BS) [33] criteria in line with the recommendations of the European Psychiatric Association guidance of the early detection of CHR-P states [2]. Details of psychopathological and neurocognitive assessments can be found in section 2.2. Exclusion criteria for all groups included contraindications for MRI and TMS [34, 35] and substance misuse in the 4 weeks before participating in the study. The CHR-P patients did not meet diagnostic criteria for psychotic disorders according to DSM-5 [36], and the HC did not have any current or past psychiatric disorders according to DSM-5 or a CHR-P status.

Demographic information is reported in Table 1 (FEP Table S1), comorbidities in supplementary Table S2. To test for significant differences between HC and CHR-P groups, Mann-Whitney U tests were calculated for age and TMS intensity, and Chi-squared tests for sex and education.

**Table 1:** Sociodemographic, psychopathological and neurocognitive data.

	CHR-P	HC	z-value/Chi-squared^1^ (df)	p-value
Total	44	58	–	–
Age	19.2 (4.2)	20.2 (3.6)	1.9	0.052
Age range (min-max)	14.5 – 30.5	14.4 – 32.3	–	–
Sex (m/f)	17/27	25/33	0.2 (1)^1^	0.650
Highest ISCED (1/2/3/5/n.a)	1/23/14/1/5	3/23/20/11/1	7.3 (3)^1^	0.064
Antipsychotic medication (y/n)	7/ 37	0/58	–	–
SIPS P1	2.6 (1.3)	0.09 (0.43)	−7.0	< 0.001
SIPS P2	2.4 (1.6)	0.02 (0.13)	−8.3	< 0.001
SIPS P3	0.5 (0.9)	0 (0)	−4.0	< 0.001
SIPS P4	3.3 (1.3)	0.66 (0.66)	−9.0	< 0.001
SIPS P5	0.8 (1.0)	0.09 (0.47)	−5.3	< 0.001
SIPS N1	1.8 (1.6)	NA	–	–
SIPS N2	2.8 (1.4)	NA	–	–
SIPS N3	2.0 (1.6)	NA	–	–
SIPS N4	3.3 (2.0)	NA	–	–
SIPS N5	0.6 (1.1)	NA	–	–
SIPS N6	2.5 (2.0)	NA	–	–
APS (y/n)	34/10	–	–	–
BIPS (y/n)	0/44	–	–	–
COPER	11.8 (9.6)	0.1 (0.4)	−9.0	< 0.001
COGDIS	8.4 (7.7)	0.0 (0.1)	−8.0	< 0.001
SOFAS	59.7 (10.2)	87.8 (4.8)	8.1	< 0.001
AVLT 1	46.9 (27.8)	70.9 (21.8)	−6.1	< 0.001
AVLT total	50.6 (33.7)	74.7 (22.5)	−9.3	< 0.001
AVLT delay	44.7 (32.9)	69.8 (22.04)	−9.3	< 0.001
DSST	10.8 (5.8)	12.6 (2.7)	−9.5	< 0.001
TMS intensity	54.2 (7.3)	53.7 (7.4)	−0.1	0.933

Sociodemographic information of CHR-P and HC groups, mean values on items scoring positive and negative symptoms, mean across items of COPER and COGDIS, current value in the SOFAS, percentiles of the AVLT first, total, and delayed recall and standardized score of the DSST of the neurocognitive test battery and mean TMS intensity applied (in percentage of the total possible output of the TMS stimulator). Negative symptoms were not assessed in HC. In case not otherwise stated, mean and standard deviation (SD, in brackets) are displayed.

*CHR-P* clinical high-risk for psychosis; *HC* healthy controls; *ISCED* International Standard Classification of Education; *SIPS* Structured Interview for Psychosis-Risk Syndromes; *APS* Attenuated Psychotic Symptoms; *BIPS* Brief Intermittent Psychotic Symptoms; *COPER* Cognitive Perceptive; *COGDIS* Cognitive Disturbances, *SOFAS* Social and Occupational Functioning Scale; *AVLT* Auditory Verbal Learning Test; *DSST* digit symbol substitution test.

Both HC and patients completed a psychopathological interview, neurocognitive tests, an MRI measurement, and a TMS-EEG session. Patients received 125.- as compensation for their participation in the study, HC 150.- CHF. Only the TMS-EEG session provided additional effort besides standard diagnostic procedures done at the FETZ Bern, which is why patients received a reduced amount.

### Psychopathological and neurocognitive assessments

Psychopathological and neurocognitive assessments included those performed at the FETZ Bern [29]. The Structured Interview for Psychosis-Risk Syndromes (SIPS) [37] was used to assess UHR, the Adult [38] or Child & Youth version of the Schizophrenia Proneness Instrument (SPI-A/SPI-CY) [39] BS criteria, which include cognitive-perceptive basic symptoms (COPER) and cognitive disturbances (COGDIS). The SIPS was rated as fulfilled by the presence of either attenuated psychotic symptoms (APS; any SIPS positive (P) items with a score between 3 and 5, beginning in the past year or scoring at least one point higher than a year prior, occurring at least once per week in past month), brief intermittent psychotic symptoms (BIPS; any P item with a score of 6, started in past three months, present minimum several minutes/day at least once a month) or genetic risk (first degree relative with any psychotic disorder and/or patient has DSM-IV diagnosis of Schizotypal Personality disorder) and functional decline criterion (30% or greater drop in global assessment of functioning in past month compared to prior 12 months). COPER was rated as fulfilled if (1) at least one of symptoms 5 to 14 was reported by the patient, if (2) the symptom first occurred more than 12 months ago, and if (3) the symptom reached a severity of three or higher. The COGDIS was rated as fulfilled if (1) at least one of symptoms 1 to 9 was reported by the patient and if (2) the symptom reached a severity of three or higher. The Social and Occupational Functioning Assessment Scale (SOFAS) [40] was used to assess level of functioning, the Mini-International Neuropsychiatric Interview (MINI) for adults [41] or for children [42] was used to exclude the presence of a psychiatric disorder in HC. HC were only assessed in the positive symptom domains of UHR criteria, which is why no scores for negative items are available.

As measures for symptoms and functioning, the positive and negative subscales of the SIPS and the SOFAS were chosen to be further analyzed because of their predictive significance for the transition to psychosis [43, 44]. The BS criteria of the SPI were also evaluated, as they are associated with more severe psychopathology [45, 46].

Among the neurocognitive tests, the German version of the Auditory Verbal Learning Test (AVLT) [47] and the Digit Symbol Substitution Test (DSST, testing processing speed) [48–50] were further analyzed. These tests have previously been shown to reveal the greatest difference between HC and CHR-P individuals [51]. Average values and results of statistical comparisons between groups are reported in Table 1.

### TMS-EEG recording and neuronavigation

The TMS stimulator (MagPro R30, Tonica Elektronik A/S, Lucernemarken, Denmark) was used with a figure-eight coil (C-B60, Tonica Elektronik A/S, Lucernemarken, Denmark). Active motor threshold (aMT) was assessed with a 64-channel EEG cap (BrainCap TMS, Brainproducts, Gilching, Germany) already mounted, by stimulating the hand area of primary motor cortex while participants sat with their arm on an armrest and index finger slightly elevated. The TMS intensity eliciting a finger twitch five out of ten times was defined as aMT.

Using a neuronavigational system (Brainsight, Rogue Research, Montréal, Canada) with individual MRI images, target regions (lDLPFC, lPPC and DMPFC) were marked on the EEG cap. MRI data was acquired on a 3.0 Tesla whole-body Siemens MRI system (Prisma, Siemens Medical Systems, Erlangen, Germany). A high-resolution magnetization-prepared rapid acquisition gradient echo (MPRAGE) T1 sequence was recorded with repetition (TR) = 2300 ms, an echo time (TE) of 2.98 ms and inversion (TI) of 900 ms, covering 240 × 257 x 160 voxels with a slice thickness of 1.0 mm and voxel size of 1 × 1 × 1 mm^3^ [52]. The individual T1 weighted MRI images were first converted from DICOM to NIFTI format with dcm2niix (https://github.com/rordenlab/dcm2niix), removing identifying information from the header. The MRI data was then further processed with a custom script in MATLAB (R2021a, MathWorks, Inc., Natick, MA, United States) utilizing the SPM12 (Wellcome Department of Cognitive Neurology, https://www.fil.ion.ucl.ac.uk/spm/) and CAT12 (Jena University Hospital, Departments of Psychiatry and Neurology, https://neuro-jena.github.io/cat/) toolboxes to mark the TMS stimulation targets. The targets were previously defined using the Montreal Neurological Institute (MNI) space coordinates at −42, 24, 50 (x, y, z) for targeting of the left DLPFC, coordinates −48, −50, 52 to target the left PPC and coordinates 0, 40, 50 for DMPFC. Using SPM’s inverse normalization, the defined targets were back-projected onto the individual T1 MRI images.

At each site, 100 single TMS pulses (110% aMT) were delivered with jittered 3-second intervals during concomitant EEG (Brainamp DC, Brainproducts, Gilching, Germany). Average TMS intensities are reported in Table 1. During TMS-EEG, participants wore in-ear noise-cancelling earbuds playing white noise to reduce auditory artifacts of the TMS coil click and protect hearing. The coil was not touching the head to further reduce bone-conducted auditory artifacts [53] and mechanical artifacts. It was tripod-mounted, elevated and positioned perpendicular and as closely as possible to the marked targets without touching the electrodes. No foam or additional damping material was used between the coil and the head. Electrode impedance was reduced below 5 kΩ if possible using electroconductive EEG gel. A sampling rate of 2500 Hz was used with FCz as the online reference and AFz as ground electrode. Automatic direct current (DC) correction was applied when signal saturation reached 75%. During the measurement, participants sat with their heads supported and eyes open, fixating a cross to minimize movement artifacts. Stimulation target order was counterbalanced by participant ID to reduce impact of wakefulness.

### Data analysis

#### EEG preprocessing

EEG data was processed offline using MATLAB (R2021a, MathWorks, Inc., Natick, MA, United States) with EEGLAB (v14.1.2; https://sccn.ucsd.edu/eeglab/index.php) [54] and tmseeg toolboxes (v5.0; https://github.com/EEGSignalProcessing/TMSEEG/releases/tag/v5.0) [55] and analyzed with custom scripts in MATLAB and RStudio (v2023.12.0 + 369; https://posit.co/downloads/). The preprocessing pipeline used has been described in our previous work [56] and can be viewed in the supplementary material. Morlet wavelet convolution and conversion to decibel (dB) was performed with custom scripts to obtain time-frequency decomposition of the signal. A large decay artifact of the TMS pulse remained for some participants, which would have affected the time-frequency decomposition of the data. Additional linear interpolation was thus performed per channel and trial from −1 to 15 ms around the TMS pulse to improve removal. The data was then bandpass filtered from 3 – 100 Hz. To extract the total oscillatory response to TMS, Morlet wavelet convolution was performed on the preprocessed TMS-EEG data. Both the cycles and the frequencies used for the decomposition were set to logarithmically increase in 30 steps, with cycles ranging from 3 to 10 and frequencies from 3 to 80 Hz. Normalization was achieved by converting the averaged trials to decibels (dB), dividing all values by the mean baseline value from −400 to −200 ms, and multiplying by 10 times its logarithm separately for each electrode, frequency, and dataset. To reduce the number of data points, we then applied temporal averaging over bins of 20 ms, computing the mean of each interval.

#### Determining frequency bands and electrodes of interest

**Fig. 1:**
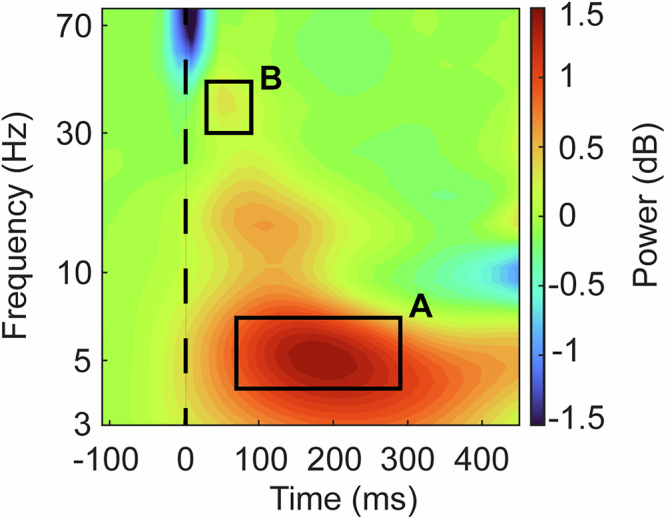
Time-frequency plot of average TMS response. Mean across all electrodes, stimulation sites and CHR-P/HC subjects (weighted by group size). Single pulse TMS produced a strong neuronal response in the theta frequency band (4 – 7.5 Hz), centering around 60 – 300 ms (**A**) and a smaller response in a low gamma frequency band (30 – 45 Hz), centering around 20 – 100 ms (**B**). These time- and frequency windows of interest are marked by the black rectangles. The dashed line represents the timing of the TMS pulse.

To define gamma and theta frequency ranges, data were first averaged across electrodes, subjects (first within and then across groups to account for different sample sizes), and conditions. Visual inspection revealed TMS-related activity in the following frequency ranges and time windows: theta (4–7.5 Hz, 60–300 ms) and gamma (30–45 Hz, 20–100 ms) (Fig. 1). According to previous studies, beta band power changes was associated with a downshift of power from gamma band range [21, 23, 26]. Beta band was only considered for further analysis if gamma power showed a significant reduction.

Electrodes of interest were identified by averaging activity over each frequency band and plotting topographies over time (Fig. S1). Theta activity was consistently localized over fronto-central sites, leading to the selection of FC1, FCz, FC2, C1, Cz, and C2. Gamma activity was more variable in its topographical distribution, thus electrodes were selected based on peak power (averaged over 20–100 ms) per stimulation site using MATLAB’s max function, resulting in C1 for lPPC and lDLPFC, and Cz for DMPFC stimulation.

#### Permutation testing

**Fig. 2:**
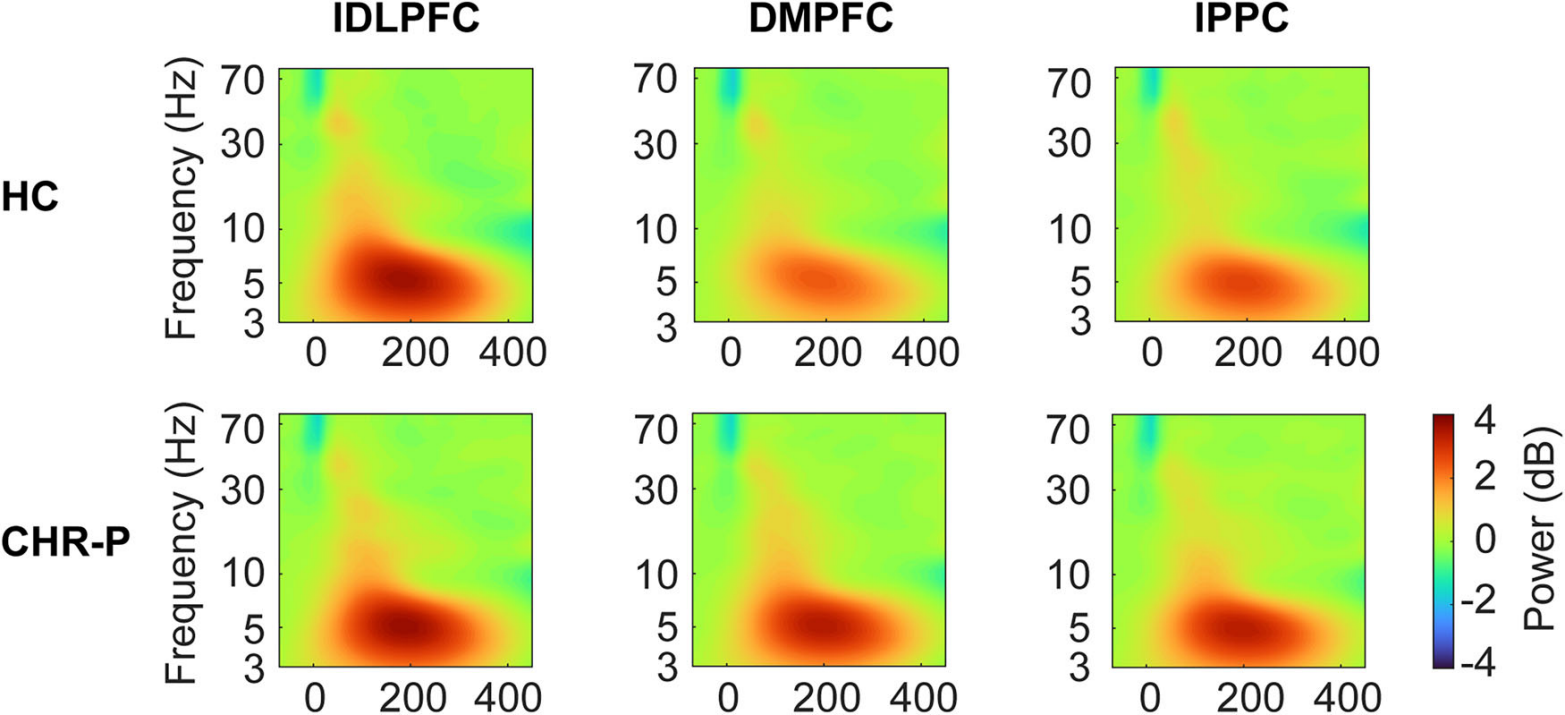
Time-frequency plots per group and stimulation site. Raw time-frequency plots of oscillatory activity for each group and TMS site separately over frontocentral sites (FC1, FCz, FC2, C1, Cz, and C2). HC (top row) show a differentiated response to TMS per stimulation location in the theta range, while CHR-P (bottom row) patients show a similar response for each TMS site.

After averaging across electrodes of interest for each group, time-frequency plots (Fig. 2) revealed a stimulation site-specific response to TMS in the theta frequency for HC, but not for CHR-P. In FEP, a similar but attenuated pattern compared to HC emerged (Fig. S2). We performed permutation tests to assess the significance of within- and between-group differences.

To test within-group variation across stimulation sites, we mean-centered the time-frequency data per TMS site and subject (for CHR-P and HC), then applied a permutation test (5000 iterations) by randomly flipping subject-level differences. Significance was evaluated using z-scores:

$$z-{map}=\frac{{mean}\left({{TF}}_{{ROI}}\right)-{mean}({permuted}\,{Differences})}{{std}({permuted}\,{Differences})}$$

We applied cluster-based correction by thresholding z-maps (p < 0.05), identifying clusters with the MATLAB function bwconncomp, and comparing them to a null distribution of maximum cluster sizes (97.5th percentile threshold), subsequently removing clusters below this threshold. Between-group comparisons were conducted per TMS site by shuffling CHR-P and HC labels and applying the same permutation and cluster correction approach. To ensure that these effects were not primarily driven by auditory-evoked or other phase-locked ERP components, we also computed induced activity by subtracting the phase-locked component from the total oscillatory response (Fig. S3).

Visual inspection identified only a small, transient gamma cluster, which was unsuitable for permutation testing due to its bias toward larger clusters. Unlike theta power, no clear differentiation between stimulated brain regions was observed, further adding to the decision not to perform permutation testing for gamma power.

#### Linear mixed effects models

To test power differences in theta and gamma frequencies among both groups and all three stimulation sites at the same time, linear mixed effect models (LMM) were calculated in R utilizing packages lme4 [57], lmerTest [58], and performance [59]. For both frequency bands, activity was first averaged across the pre-defined frequency ranges, time windows, and electrodes per subject. With the extracted theta- and gamma-band data, LMMs were fitted in R, testing the interaction between group and region for both frequency bands separately while controlling for age, antipsychotic medication, and random subject effects. As mood disorders are also associated with alterations in theta-band oscillatory activity [60], we fitted LMMs in R within the CHR-P group for both theta- and gamma-band activity separately, testing the interaction between comorbid mood disorder and region while controlling for age, antipsychotic medication, and random subject effects.

#### Association with psychopathology and neurocognition

The extracted frequency data was used to test associations with psychopathology and neurocognition within the CHR-P group. Spearman’s rank correlations were calculated separately between theta and gamma power values of each stimulation site and items P1 to P5 and N1 to N6 of the SIPS, COPER, COGDIS of SPI, the current value of the SOFAS, percentiles of the first, total, and delayed recall of the AVLT, and the standardized score of the DSST. To correct for multiple testing, false discovery rate (FDR) correction was applied using the fdr_bh function in MATLAB (https://ch.mathworks.com/matlabcentral/fileexchange/27418-fdr_bh). Missing data were handled by pairwise deletion.

## Results

The HC and CHR-P groups were statistically compared across sociodemographic, neurocognitive, and psychopathological variables to assess potential differences (Table 1). No significant differences were found in sociodemographic variables or average TMS intensities (p > 0.05). The two groups differed significantly in SIPS positive symptom scores and performance on neurocognitive tests (AVLT and DSST), with CHR-P patients having more severe positive symptoms (p < 0.001) and poorer cognitive performance (p < 0.05).

### Permutation tests of TMS-related theta activity

Based on the raw oscillatory response to TMS, a visually recognizable difference was present in HC in the theta band between stimulation locations, suggesting regional specificity (Fig. 2, top). In the CHR-P group however, the amount of theta power seemed to show no difference between stimulation locations (Fig. 2, bottom). In FEP, a difference in theta power seemed to emerge, but with decreased power (Fig. S2). Induced activity for both CHR-P and HC showed a comparable spectral-temporal pattern to the total oscillatory response, suggesting that the results are not solely driven by phase-locked ERP components (Fig. S3).

**Fig. 3:**
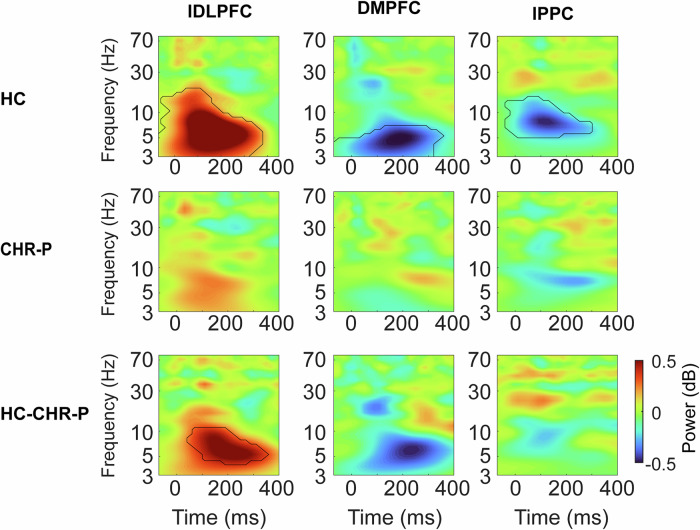
Permutation test results of differential theta activity. Time-frequency plots as the difference between activity from a specific stimulation site (lDFPC, DMPFC, lPPC) and the mean of all sites for HC (top row) and CHR-P (middle row), as well as between groups (bottom row). Power is shown as average over fronto-central sites (FC1, FCz, FC2, C1, Cz, and C2). For the top two rows, red means that activity was higher after stimulating this site compared to the mean across all TMS sites, whereas blue means lower activity compared to the mean. For the bottom row, red signifies more positive deviation from the mean in HC compared to CHR-P, whereas blue means more negative deviation. Significant clusters of activity from permutation testing are marked in black.

To formally test this regional specificity in TMS-related activity, we performed permutation testing within and between HC and CHR-P groups. The permutation tests revealed that in the HC group, theta oscillations significantly varied from the average response between all stimulation locations (Fig. 3, top). For lDLPFC stimulation, the maximum deviation from the mean across all TMS sites within the HC group was 0.80 dB at 5.92 Hz and 160 – 180 ms (*z* = 4.83, *p* < 0.001), for DMPFC stimulation - 0.51 dB at 4.72 Hz and 180 – 200 ms (*z* = −3.56, *p* < 0.001) and lPPC - 0.47 dB at 7.42 Hz and 120 – 140 ms (*z* = - 0.47, *p* < 0.001). The theta activity of CHR-P subjects did not vary significantly from the mean activation across all TMS sites (all p > 0.05) (Fig. 3, middle). Testing the groups against each other resulted in a significant cluster for the lDLPFC stimulation reaching a maximum difference between CHR-P and HC of 0.55 dB at 5.16 Hz and 220 – 240 ms (*z* = 2.67, *p* = 0.008) (Fig. 3, bottom). The results suggest that a regional differentiation of TMS-related activity in the theta band disappeared in the CHR-P group.

### Linear mixed effects models

LMMs were conducted to examine the effects of group (HC, CHR-P), TMS site (lDLPFC, lPPC, DMPFC), and age, separately for average theta and gamma frequencies, using subjects as a random effect. The full results are reported in supplementary Tables S3 and S4, extracted theta and gamma power are plotted in Figs. S4 and S5.

For TMS-related theta activity (Table S3), there was a significant main effect of TMS site in HC, indicating that average theta power was significantly lower in the DMPFC (*β* = −1.03, *SE* = 0.19, *t*(200) = −5.42, *p* < 0.001) and lPPC (*β* = −0.92, *SE* = 0.19, *t*(200) = −4.82, p < 0.001) compared to the lDLPFC. Main effects for group, age, and antipsychotic medication were not significant (*p* > 0.05). A significant group x TMS site interaction was found for DMPFC stimulation (*β* = 0.81, *SE* = 0.29, t(200) = 2.80, *p* = 0.006), indicating higher average theta power in CHR-P compared to HC for this TMS site. The group x TMS site interaction for lPPC similarly indicated higher theta power compared to HC, however this association only showed a trend toward significance (*β* = 0.55, *SE* = 0.29, *t*(200) = 1.91, *p* = 0.057). The full model, including fixed and random effects, explained 64.6% of the variance (*Conditional R²* = 0.646). These results show a loss of regional differentiation in the CHR-P group, characterized by increased TMS-related theta activity.

For TMS-related gamma activity, none of the investigated effects and interactions reached a level of significance (all *p* > 0.05) (Table S4). The full model, including fixed and random effects, explained 60.1% of the variance (*Conditional R*^*2*^ = 0.601). Because of the absence of significant differences in the gamma band, no analyses were performed on beta-band activity.

LMMs were also conducted to examine the effects of comorbid mood disorders within the CHR-P group, TMS site (lDLPFC, lPPC, DMPFC), and age, separately for average theta and gamma frequencies, using subjects as a random effect. The results are reported in supplementary Tables S5 and S6. None of the investigated effects and interactions reached a level of significance (all *p* > 0.05). The full model for theta, including fixed and random effects, explained 59.1% of the variance (*Conditional R*^*2*^ = 0.591), the full model for gamma 53.7% (*Conditional R*^*2*^ = 0.537).

### Association with psychopathology and neurocognition

In CHR-P patients, Spearman rank correlations were calculated between gamma/theta power and positive and negative symptoms, as well as neurocognition, to evaluate whether these are associated. Correlation coefficients for the associations between gamma/theta and psychopathology are reported in Table 2.

**Table 2:** Correlation coefficients for gamma/theta association with psychopathology.

		*n*	*M*	*SD*	lDLPFC	lPPC	DMPFC
Theta							
	P1	44	2.64	1.3	−0.29	−0.45**	−0.39**
	P2	44	2.43	1.54	0.02	−0.13	−0.07
	P3	44	0.48	0.89	0.01	0.1	−0.1
	P4	44	3.34	1.26	0.09	−0.07	0.12
	P5	44	0.75	1.00	0.01	−0.09	−0.13
	N1	42	1.79	1.61	0.02	0.14	0.00
	N2	42	2.79	1.42	−0.31*	−0.35*	−0.21
	N3	42	2.02	1.60	0.00	0.06	0.12
	N4	42	3.29	1.97	−0.32*	−0.45**	−0.28
	N5	42	0.64	1.13	−0.13	−0.27	−0.07
	N6	42	2.48	1.94	−0.46**	−0.44**	−0.25
Gamma							
	P1	44	2.64	1.30	0.45**	0.17	0.03
	P2	44	2.43	1.54	0.17	0.11	0.04
	P3	44	0.48	0.89	0.18	−0.16	−0.14
	P4	44	3.34	1.26	0.00	−0.06	−0.33*
	P5	44	0.75	1.00	0.01	0.09	−0.01
	N1	42	1.79	1.61	−0.05	0.01	−0.21
	N2	42	2.79	1.42	0.01	0.04	−0.14
	N3	42	2.02	1.60	0.13	0.13	0.00
	N4	42	3.29	1.97	0.04	−0.01	−0.01
	N5	42	0.64	1.13	0.09	0.02	0.02
	N6	42	2.48	1.94	0.01	−0.12	−0.07

Result of the Spearman rank correlation between TMS-related gamma/theta power and psychopathological item scores. P1 – P5 represent positive and N1 – N6 negative symptoms in the SIPS. Significant correlations surviving false discovery rate (FDR) correction are depicted in bold font. Uncorrected significant p-values are indicated by asterisks (*p* ≤ 0.05, ** *p* ≤ 0.01, *** *p* ≤ 0.001). Corresponding FDR-corrected p-values ranged from 0.01 to 0.05.

Theta power after lDLPFC (*M* = 2.88, *SD* = 1.71) and lPPC stimulation (*M* = 2.52, *SD* = 1.52) negatively correlated with SIPS N2 (avolition), N4 (decreased experience of emotion) and N6 (deterioration in role functioning). Theta power after lPPC and DMPFC stimulation (*M* = 2.66, *SD* = 1.83) was negatively associated with SIPS P1 (unusual thought content). Among these correlations, the associations between lDLPFC theta and N6, lPPC theta and P1, N4, and N6, as well as lDLPFC gamma and P1 survived FDR correction (*p*(FDR) < 0.05, Table 2).

Gamma power after lDLPFC stimulation (*M* = 0.93, *SD* = 0.83) was positively correlated with SIPS item P1 (unusual thought content) and negatively after DMPFC stimulation (*M* = 0.84, *SD* = 1.17) with item P4 (perceptual abnormalities).

Neither gamma nor theta power was significantly (*p* > 0.05) associated with BS criteria (COPER and COGDIS), SOFAS score, or the neurocognitive tests evaluated (supplementary Tables S7 – S10).

## Discussion

The aim of the present study was to investigate differences between CHR-P patients and HCs in the oscillatory cortical response to single-pulse TMS measured by EEG and associations to neurocognitive and psychological assessments. The time-frequency analysis demonstrated that TMS stimulation resulted in different intensities of the theta oscillatory response over fronto-central electrodes in the HC group depending on the site of stimulation. Targeting the lDLPFC produced the largest response, followed by lPPC and DMPFC. In contrast, this differentiation was absent in the CHR-P group, and the oscillatory response to all stimulation sites was comparable to lDLPFC stimulation in HC. In the FEP group (Fig. S2), where due to limited sample size only visual inspection was conducted, differences between regions were observable, but with attenuated power compared to HC.

As the HC showed a varying response depending on stimulation location, we interpret these results as depicting a functional specialization of the stimulated brain regions. It has been widely accepted that different regions in the brain and their networks serve different functions, and thus show a “functional fingerprint”, which might be based on varying amounts of connections in the brain or the “connectional fingerprint” [61]. Theta band oscillations have been linked to long-range communication [17], and the increase and decrease in theta power after TMS stimulation suggests more or less synchronized activity of neurons depending on the stimulated area. The absence of such a differentiated response to TMS in the theta frequency range in our CHR-P patients hints at a possible loss of functional specialization, which could be due to developmental impairments of the “connectional fingerprint” observed in healthy individuals.

Testing associations with the positive and negative items of the SIPS in CHR-P, averaged theta power of lDLPFC and lPPC stimulation was negatively correlated with three negative items (avolition, decreased experience of emotion, and deterioration in role functioning), while theta after lPPC and DMPFC stimulation was negatively correlated with one positive item (unusual thought content). These negative associations imply that higher power was associated with less severe symptoms, reflecting a compensatory functional mechanism for possible structural grey and white matter impairments [12, 13]. This compensation leads to a loss of functional specialization, with all stimulated brain regions reacting in a similar manner to TMS stimulation. It is possible that once this compensation fails, CHR-P individuals convert to a first psychotic episode. According to Dynamical Systems Theory, this transition can be understood as a “tipping point”, a critical shift in the brain’s dynamics [62, 63]. As the brain undergoes gradual structural and functional changes during the trajectory from CHR-P to FEP, these may initially be compensated for by recruitment of additional networks or brain structures. However, when a tipping point is reached, the compensatory mechanisms are no longer sufficient, the brain’s stability is compromised, and small perturbations can trigger a conversion to psychosis. We hypothesize such a mechanism based on the observation that the FEP group shows a differentiated theta pattern comparable to HC, but with diminished power. Indeed, a reduction in theta power among FEP individuals is consistent with previous studies reporting diminished theta power following frontal [27] and motor cortex [24] stimulation compared to HC. However, no definite conclusions can be drawn due to the very small sample size in the current FEP group.

This proposed compensatory mechanism might not be exclusive to schizophrenia and its high-risk state, but rather represents a general biological response to structural decline. A similar mechanism is suggested in neurodegenerative diseases like Alzheimer’s [64]. An increase in functional connectivity and reorganization paired with structural decline was found in people with mild cognitive impairment (MCI), a prodromal state of dementia, supporting the notion of a compensatory mechanism [65, 66]. The transition from MCI to dementia may mark a tipping point where compensatory processes begin to fail [67]. In schizophrenia, these mechanisms may persist: fMRI studies show that under high working memory load, task-unrelated brain regions and connections are aberrantly recruited, indicating a loss of functional specialization as a trade-off for compensation [68–70]. Interestingly, a recent study in children and adolescents with first-episode psychosis reported that theta responses during an oddball task followed the pattern HC < CHR-P < FEP, indicating that theta is elevated both in the high-risk state and in FEP [71]. In contrast, our TMS–EEG results show decreased theta in FEP at targeted stimulation sites. This difference may reflect the distinction between paradigms: task-based designs capture theta generated through compensatory recruitment of task-unrelated regions, whereas TMS–EEG probes local circuit responsiveness, which may already be compromised in FEP.

Although the correlations between oscillatory power and clinical symptoms may appear inconsistent at first, they can be interpreted in the context of network-level and compensatory mechanisms. Theta power increases following lDLPFC and lPPC stimulation were associated with reduced severity of avolition, decreased experience of emotion, and deterioration in role functioning. The FPN, which includes the DLPFC and PPC, supports cognitive control and goal-directed behavior [72]. Impairments in these domains could manifest in the form of avolition as well as deterioration in role functioning. In addition, both the DLPFC and parietal cortex contribute to cognitive reappraisal during emotion regulation [73], providing a plausible link to reduced emotional experience. The only positive symptom significantly correlated with theta power after DMPFC and lPPC stimulation was unusual thought content. Functionally, the PPC contributes to salience detection [74], while the DMPFC is involved in self-referential processing [75]. Aberrant attribution of significance to neutral stimuli or internal thoughts, combined with altered self-referential processing, could contribute to unusual thought content.

It has been suggested that the N100 and P200 components of TMS-evoked potentials (TEPs) are the main contributors to the TMS-related theta response [76], which largely reflects an auditory response to the TMS pulse [53, 77, 78]. Although noise-cancelling headphones and white noise were used during TMS, participants still reported hearing the coil click. The coils’ position, varying by stimulation site, might slightly alter the sound’s auditory property and produce different theta responses; however, this does not explain the lack of such a differentiation in the CHR-P group. Additionally, according to a review article by Hamilton and colleagues [79], multiple studies evaluating N100 and P200 components of auditory evoked potentials have found reductions in amplitude in both psychotic and CHR-P patients compared to HCs, which would be reflected in a generally reduced oscillatory response in CHR-P. Thus, the regional specificity in HC and its absence in CHR-P cannot be explained by sensitivity to auditory stimuli alone. Supporting this, time-frequency plots of induced activity (Fig. S3) showed similar patterns, suggesting that the effects are not solely driven by phase-locked components such as an auditory artifact. While auditory artifacts may still influence the theta responses, several factors suggest a genuine neural basis for absence of theta-differentiation in CHR-P. These include the presence of inter-regional variability in the HC group (absent in CHR-P), the direction of N100/P200 effects relative to prior auditory studies, and similar patterns in induced activity as observed in the total activity (Fig. S3).

Previous studies evaluating TMS-EEG measures in schizophrenia patients have consistently found reductions in the oscillatory response in the gamma frequency range [21–26], which seemed to be due to a downshift from gamma to beta in the natural oscillatory response [21, 23, 26]. In the present study, we found no significant differences in the gamma band between the groups. Interestingly, gamma after lDLPFC stimulation was positively associated with one SIPS positive symptom (unusual thought content) and negatively with another positive symptom (perceptual abnormalities) after DMPFC stimulation. These findings are hard to interpret as the associations point in opposing directions. For theta oscillations, we observed consistent negative correlations across areas and associated SIPS negative symptoms. For gamma oscillations, however, the direction of significant correlations was rather stochastic. This finding might therefore be incidental and needs further confirmation to interpret.

The absence of significant differences between HC and CHR-P groups in the gamma response may have several explanations. First, gamma oscillations are thought to represent localized neural communication [17]. The immediate cortical response to TMS at the stimulation location happens at latencies up to 10 ms, after which the signal spreads contralaterally [19]. Due to remaining decay artifacts of the TMS pulse, data up until 15 ms post-pulse had to be removed, possibly removing early gamma components. This aligns with gamma activity being observed at central, not stimulation-site electrodes. It is also possible that the slowing of high-frequency oscillations as observed in other studies [23, 26] might happen gradually from prodrome to chronic disease and possibly only reach statistical significance at frank psychotic onset. Notably, such frequency shifts are not unique to schizophrenia and have also been reported in bipolar disorder and major depression [21].

### Limitations

The small sample size of the FEP patient group limited the feasibility of statistical comparisons. Furthermore, the data were only cross-sectional, preventing conclusions about disease progression and possible transition to FEP and their effects on TMS-EEG parameters. Longitudinal data would allow us to draw conclusions on whether the changes in oscillatory response to TMS in the CHR group are indeed compensatory, and if a breakdown of these mechanisms marks the transition to a first psychotic episode. Additionally, it could be determined whether alterations in the gamma frequency bands only occur in full-blown psychotic disorder or if the absence of results was due to methodological constraints such as excessive TMS artifacts. Finally, our sample size was limited (n = 44), and with an expected conversion rate of 25%–35% [3], only a small subset of participants would be expected to develop psychosis, limiting the statistical power to detect effects specifically related to transition. Future studies should aim to include sufficient numbers of both CHR-P and FEP or adopt a longitudinal study design.

Additionally, to further rule out auditory artifacts as a contributor to the observed effects in the theta frequency range, future studies could include sham stimulation conditions, such as turning the coil away from the head. The resulting auditory response could help to isolate the neural responses to TMS.

## Conclusion

To our knowledge, this is the first study evaluating TMS-EEG parameters in CHR-P patients. The inclusion of three different stimulation sites enabled us to not only conduct between-group comparisons but also to evaluate differences in responses across those sites. In healthy individuals, the fronto-central oscillatory response to TMS in the theta frequency varied between different cortical stimulation sites. In the CHR-P group, this variability was absent because of increased theta-range responses, indicating a loss of functional specialization. The increased theta-range responses in CHR-P were inversely correlated with certain positive and negative symptoms, suggesting a possible compensatory mechanism against psychosis onset. We conclude that TMS-EEG has the potential to improve the understanding of psychosis risk.

## Supplementary information

Supplementary Material

## Data Availability

Data supporting the findings of this study are available from the corresponding author, upon reasonable request.

## Code availability

All code used to generate results in this paper can be made available upon reasonable request to the authors. Software versions are reported above.
