# Supplementary material for: Loss of regional theta differentiation in TMS-EEG response marks network dysfunction in psychosis risk
Source: Transl Psychiatry. 2026 Apr 10;16:255. doi: 10.1038/s41398-026-04030-5 (PMC13184105; doi:10.1038/s41398-026-04030-5)
Supplement: Supplementary file 1 — Supplementary Material [file 41398_2026_4030_MOESM1_ESM.docx]

**Supplementary material for**

Loss of regional theta differentiation in TMS-EEG response marks network dysfunction in psychosis risk

Authors: Nadja Zimmermann, Matthias Liebrand, Chantal Michel, Miriam Stüble, Arndt-Lukas Klaassen, Eva Burkhardt, Roland Wiest, Michael Kaess, Jochen Kindler & Yosuke Morishima

This file contains

Supplementary methods

Supplementary Figures S1 – S5

Supplementary Tables S1 – S10

Supplementary references

**Supplementary methods**

EEG preprocessing

The following description of our preprocessing pipeline is a copy from our previous publication for sake of completeness (1):

TMS-EEG data were segmented into epochs from -500 to 500 ms in relation to the TMS pulse, and baseline was set to -200 to -2 ms. Mastoid channels (TP9 and TP10) were removed due to excessive muscle artifacts. Data were manually inspected and epochs with excessive noise and eye blinks in the time period -200 to 400 ms relative to the TMS pulse removed. An average number of 78.6 and 75.2 epochs were kept for HC and CHR groups respectively from the 100 single-pulse TMS epochs for further analyses. If a channel showed electrode artifacts or if excessive decay of the TMS artefact was localized to one channel, it was interpolated. The reference was set to the average reference. Then to remove the initial TMS artifact and its associated decay, the TMS pulse was cut with the tmseeg toolbox from -1 to 7 ms around the TMS pulse and leftover decay was removed by Independent Component Analysis (ICA). The missing 8 ms of data was later linearly interpolated. Some participants produced very small blink artifacts right after the TMS pulse, in addition to regular blinks at later timepoints. When the larger regular blinks—which usually occurred at later time points after 500 ms—coexist with the smaller blinks within epoched data, the immediate small TMS-induced blinks were not effectively distinguished from other brain activities by ICA. As a result, we chose to cut the epochs from -500 to 500 ms relative to TMS onset to exclude the late larger blinks, which improved the removal of the smaller blinks by ICA.

Table S1: Sociodemographic, psychopathological and neurocognitive data

|  | FEP |
| --- | --- |
| Total | 9 |
| Mean age | 17.3 (2.7) |
| Age range (min-max) | 14.4 – 22.9 |
| Sex m/f | 4/5 |
| Highest ISCED (1/2/3/5/n.a) | 0/1/4/0/4 |
| Antipsychotic medication y/n | 5/9 |
| SIPS P1 | 5.0 (1.2) |
| SIPS P2 | 3.6 (2.6) |
| SIPS P3 | 2.0 (2.8) |
| SIPS P4 | 3.5 (2.7) |
| SIPS P5 | 1.8 (2.6) |
| SIPS N1 | 1.8 (2.2) |
| SIPS N2 | 2.2 (1.9) |
| SIPS N3 | 1.5 (2.1) |
| SIPS N4 | 3.0 (2.5) |
| SIPS N5 | 0.7 (1.0) |
| SIPS N6 | 4.2 (1.3) |
| COPER | 11.9 (14.3) |
| COGDIS | 7.8 (9.0) |
| SOFAS | 59.5 (17.5) |
| AVLT 1 | 55.6 (30.6) |
| AVLT total | 51.1 (35.0) |
| AVLT delay | 42.5 (37.1) |
| DSST | 8.0 (2.1) |
| TMS intensity | 53.3 (4.6) |

*Note.* Sociodemographic information of FEP group, mean values on items scoring positive and negative symptoms, mean across items of COPER and COGDIS, current value in the SOFAS, percentiles of the AVLT and standardized score of the DSST of the neurocognitive test battery and mean TMS intensity applied (in percentage of the total possible output of the TMS stimulator). Standard deviations are reported in brackets. Abbreviations: FEP = First Episode Psychosis; ISCED = International Standard Classification of Education; SIPS = Structured Interview for Psychosis-Risk Syndromes; COPER = Cognitive Perceptive; COGDIS = Cognitive Disturbances, SOFAS = Social and Occupational Functioning Scale; AVLT = Auditory Verbal Learning Test); DSST = digit symbol substitution test.

**Table S2: Comorbidities in CHR-P and FEP**

|  | F00-F09 | F10-F19 | F30-F39 | F40-F48 | F50-F59 | F60-F69 | F80-F89 | F90-F98 |
| --- | --- | --- | --- | --- | --- | --- | --- | --- |
| CHR-P | 6 | 3 | 14 | 14 | 2 | 1 | 2 | 4 |
| FEP | 0 | 0 | 3 | 2 | 0 | 0 | 0 | 0 |

*Note.* Amount of patients with comorbidities according to ICD-10 categories (2): F00-F09: Organic, including symptomatic, mental disorders; F10-F19: Mental and behavioural disorders due to psychoactive substance use; F30-F39: Mood [affective] disorders; F40-F48: Neurotic, stress-related and somatoform disorders; F50-F59: Behavioural syndromes associated with physiological disturbances and physical factors; F60-F69: Disorders of adult personality and behaviour; F80-F89: Disorders of psychological development; F90-F98: Behavioural and emotional disorders with onset usually occurring in childhood and adolescence.

**Fig. S1: Topographic plots across groups per stimulation site and frequency band**

**
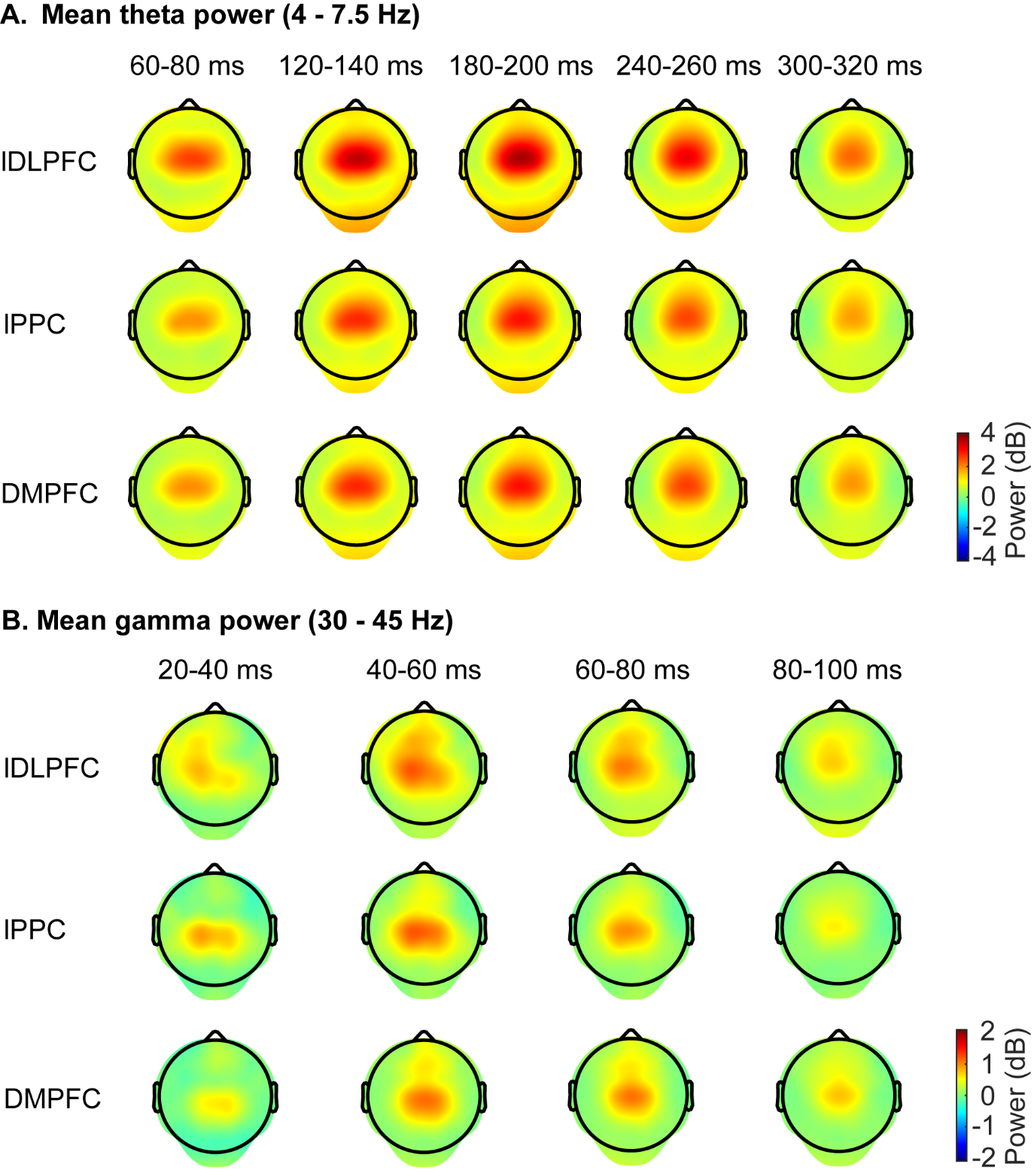
**

Topographic plots of the averaged power over HC and CHR-P groups per stimulation site and frequency band. (A) Plot of every third time bin for averaged theta power (4 – 7.5 Hz) from 60 – 320 ms. (B) Plot of averaged gamma power (30 – 45 Hz) from 20 – 100 ms.

**Fig. S2: Time-frequency plots per group and stimulation site.**


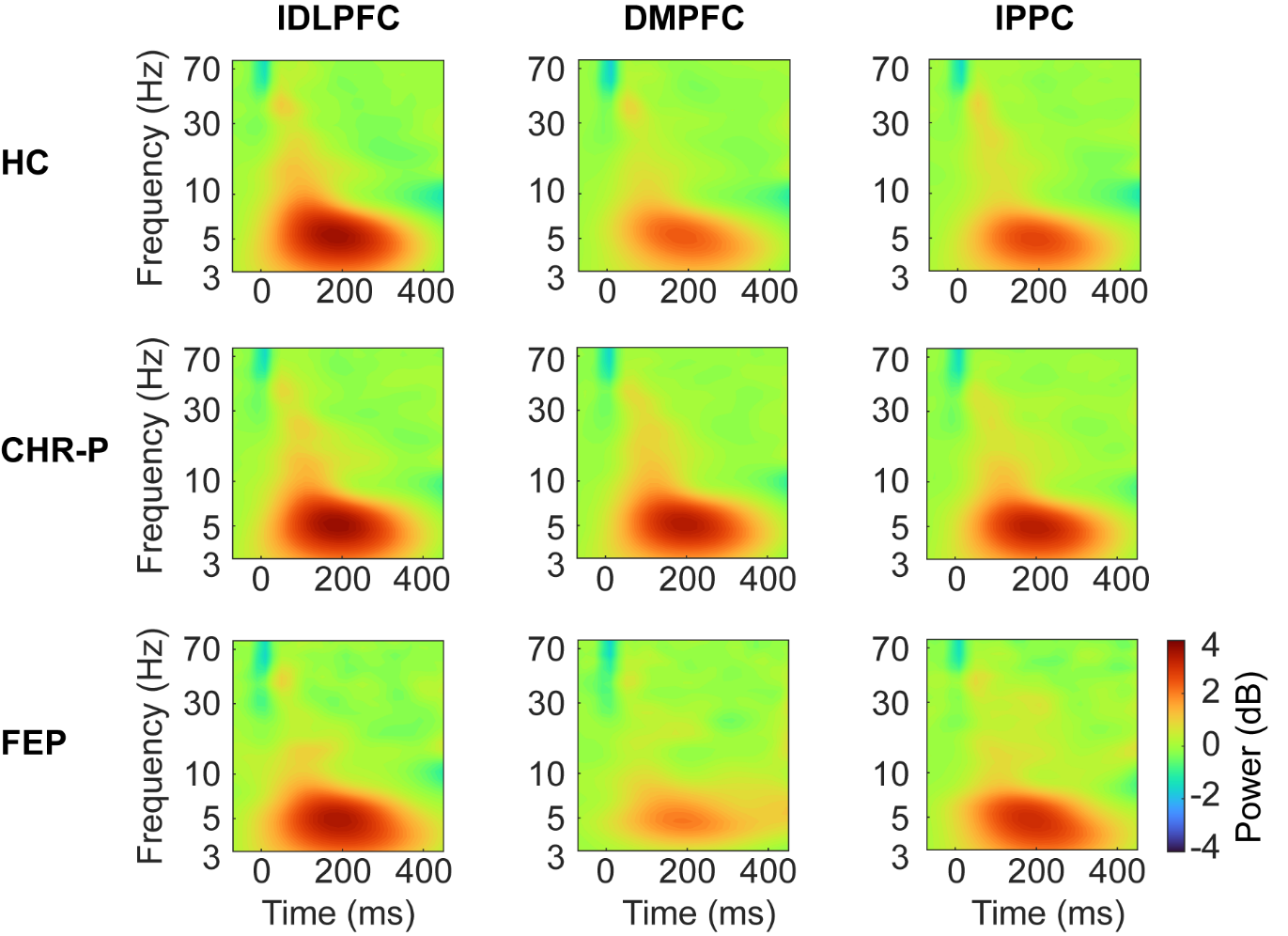


Raw time-frequency plots of theta activity for each group and TMS site separately. The healthy control (HC, top row) group shows a differentiated response to TMS per stimulation location in the theta range, while clinical high risk (CHR, middle row) patients shows a similar response for each TMS site. The first episode psychosis patients (FEP) display a differentiated theta response similar to the HC but with attenuated power.

**Fig. S3: Time-frequency plots of non-phase-locked differential theta activity**

**
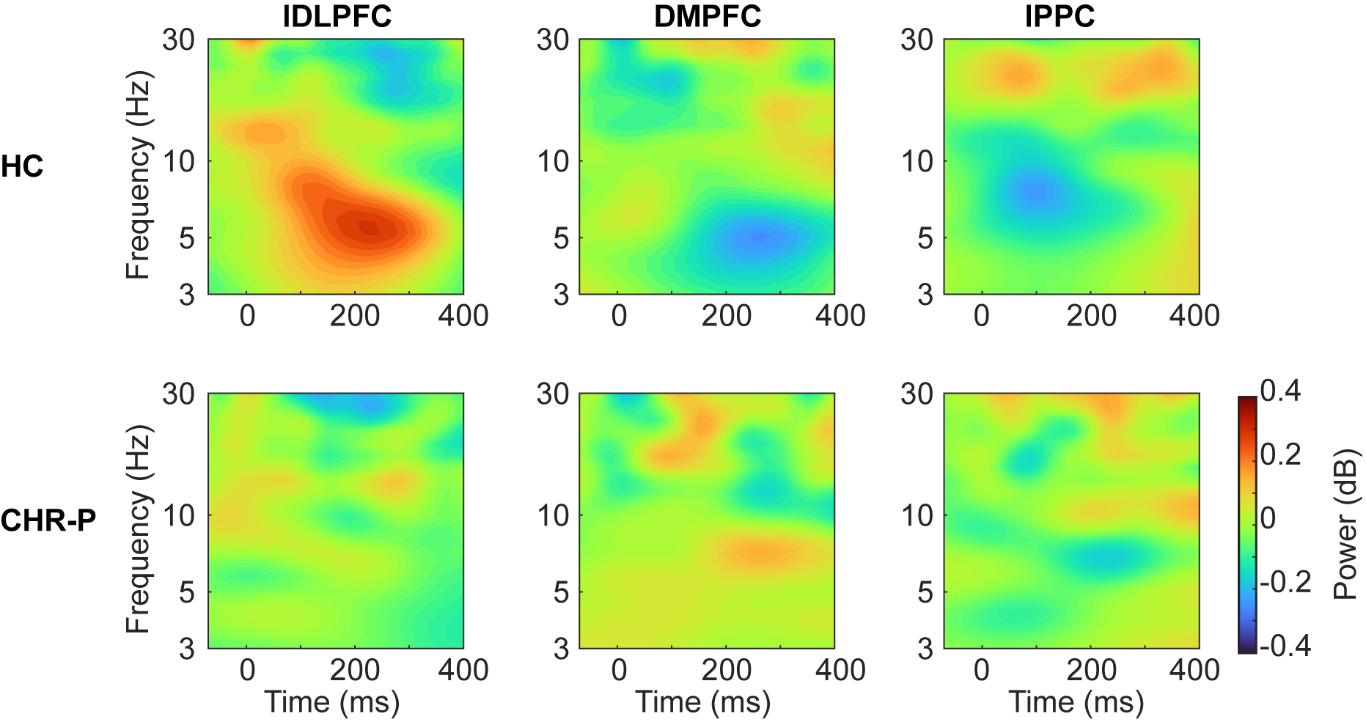
**

Time-frequency plots of TMS-induced power depicting the difference between the

activity from one stimulation site (left DLPFC, DMPFC, left PPC) and the mean across all sites. HC are depicted in the top row and CHR-P in the bottom row. Power is shown as the average over fronto-central sites (FC1, FCz, FC2, C1, Cz, and C2).

**Fig. S4: Average theta responses to TMS.**

**
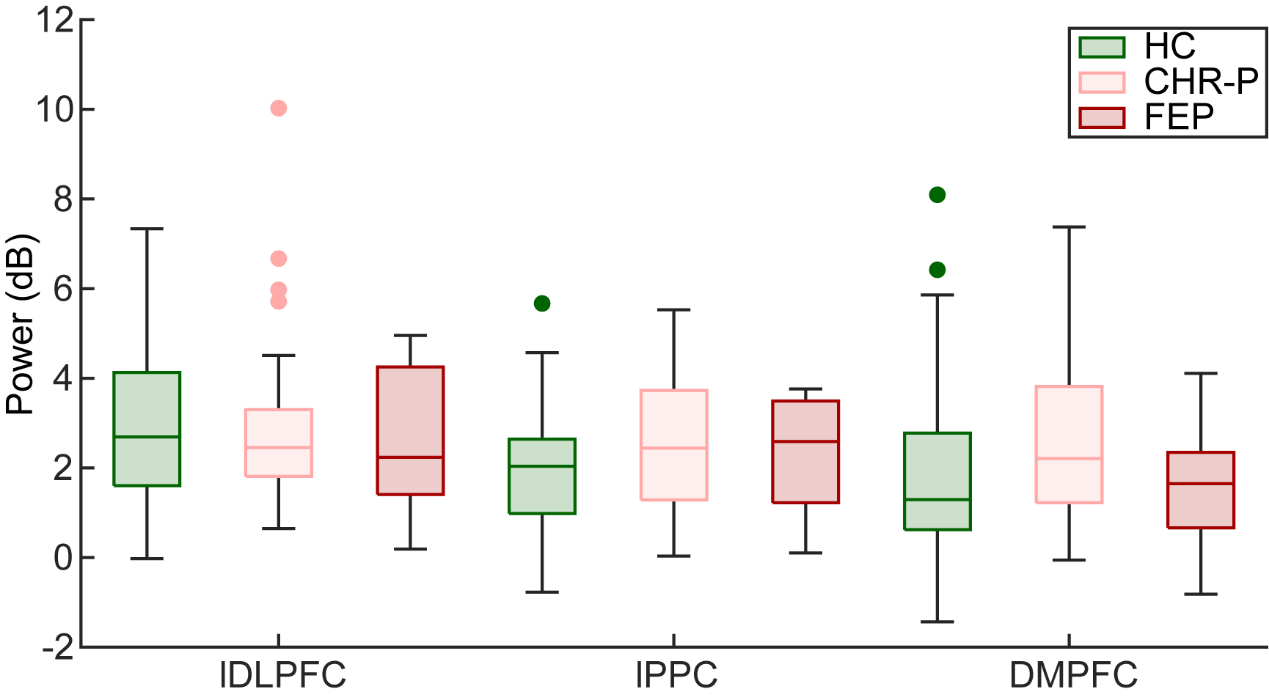
**

Boxplot of averaged theta power, grouped by region and group. The line in the boxes represents the median, whereas the whiskers extend to the smallest and largest non-outlier values. Outliers are represented as dots and are categorized as such if they are 1.5 interquartile ranges away from the 0.75 or 0.25 quantile.

**Fig. S5: Average gamma responses to TMS.**

**
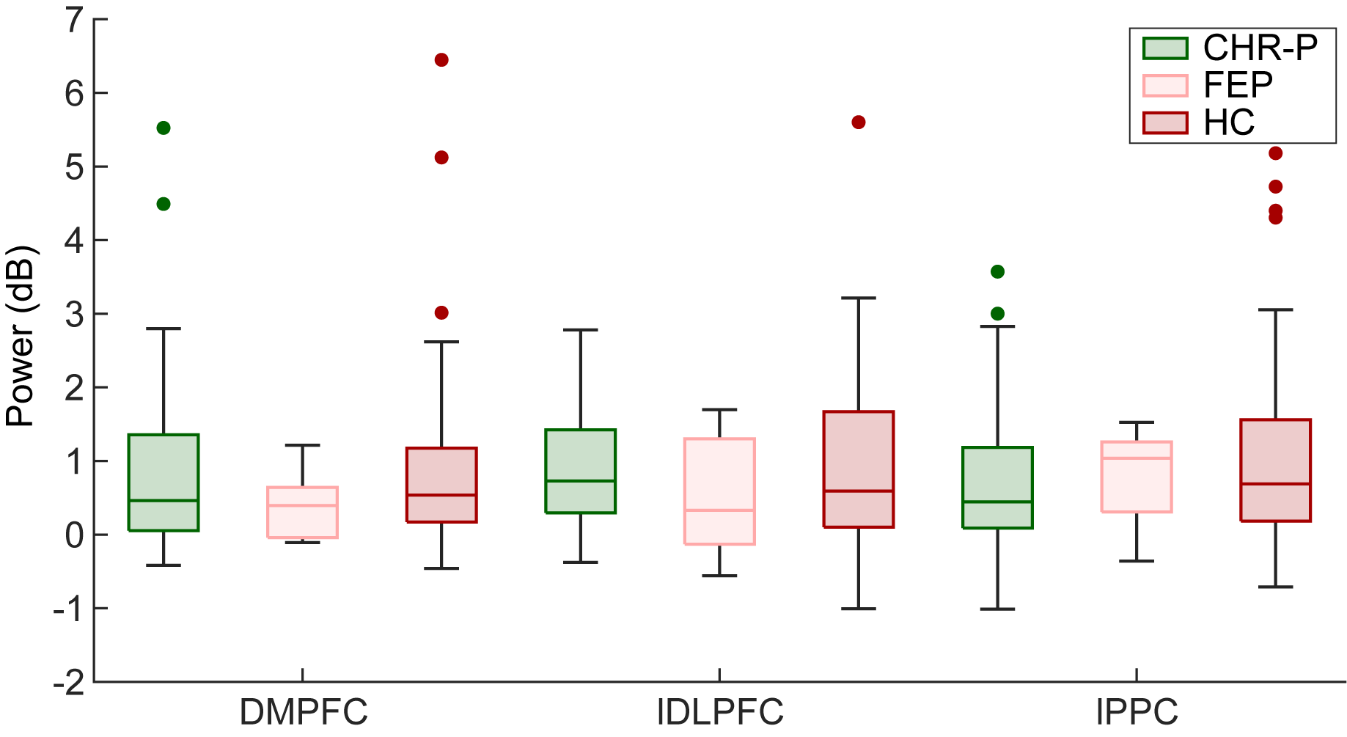
** Boxplot of averaged gamma power, grouped by region and group. The line in the boxes represents the median, whereas the whiskers extend to the smallest and largest non-outlier values. Outliers are represented as dots and are categorized as such if they are 1.5 interquartile ranges away from the 0.75 or 0.25 quantile.

**Table S3: Linear mixed effect model for theta power**

|  | Estimate | SE | df | t-value | p |  |
| --- | --- | --- | --- | --- | --- | --- |
| HC lDLPFC (Intercept) | 3.08 | 0.77 | 102.04 | 3.98 | 0.000 | *** |
| CHR-P | 0.11 | 0.35 | 159.56 | 0.31 | 0.760 |  |
| DMPFC | -1.03 | 0.19 | 200.00 | -5.42 | 0.000 | *** |
| lPPC | -0.92 | 0.19 | 200.00 | -4.82 | 0.000 | *** |
| Age | -0.01 | 0.04 | 98.00 | -0.23 | 0.819 |  |
| Antipsychotic medication | -0.91 | 0.59 | 98.00 | -1.55 | 0.124 |  |
| CHR-P x DMPFC | 0.81 | 0.29 | 200.00 | 2.80 | 0.006 | ** |
| CHR-P x lPPC | 0.55 | 0.29 | 200.00 | 1.91 | 0.057 | . |

*Note*. Results of the linear mixed effect model for theta power. The intercept represents the reference group (HC) and reference region (lDLPFC). Subject was used as a random effect. Abbreviations: SE = standard error; df = degrees of freedom.

. p ≤ .1, * p ≤ .05, ** p ≤ .01, *** p ≤ .001

**Table S4: Linear mixed effect model for gamma power**

|  | Estimate | SE | df | t-value | p |
| --- | --- | --- | --- | --- | --- |
| HC lDLPFC (Intercept) | 0.28 | 0.54 | 102.39 | 0.52 | 0.602 |
| CHR-P | -0.01 | 0.24 | 164.73 | -0.04 | 0.968 |
| DMPFC | -0.03 | 0.14 | 200.00 | -0.20 | 0.844 |
| lPPC | 0.07 | 0.14 | 200.00 | 0.48 | 0.634 |
| Age | 0.03 | 0.03 | 98.00 | 1.28 | 0.204 |
| Antipsychotic medication | 0.23 | 0.41 | 98.00 | 0.56 | 0.579 |
| CHR-P x DMPFC | -0.06 | 0.21 | 200.00 | -0.31 | 0.757 |
| CHR-P x lPPC | -0.28 | 0.21 | 200.00 | -1.32 | 0.188 |

*Note*. Results of the linear mixed effect model for gamma power. The intercept represents the reference group (HC) and reference region (lDLPFC). Subject was used as a random effect. Abbreviations: SE = standard error; df = degrees of freedom.

**Table S5: Linear mixed effect model for theta power** **including mood disorders**

|  | Estimate | SE | df | t-value | p |  |
| --- | --- | --- | --- | --- | --- | --- |
| Intercept | 2.28 | 1.06 | 42.32 | 2.15 | 0.038 | * |
| DMPFC | -0.47 | 0.31 | 84 | -1.54 | 0.128 |  |
| lPPC | 0.01 | 0.31 | 84 | 0.02 | 0.985 |  |
| MD | -0.04 | 0.53 | 73.25 | -0.07 | 0.944 |  |
| Age | 0.04 | 0.05 | 40 | 0.68 | 0.498 |  |
| Antipsychotics | -0.91 | 0.60 | 40 | -1.52 | 0.137 |  |
| DMPFC x MD | 0.36 | 0.49 | 84 | 0.72 | 0.471 |  |
| lPPC x MD | -0.07 | 0.49 | 84 | -0.15 | 0.883 |  |

*Note*. Results of the linear mixed effect model for theta power. The intercept represents the reference group (no comorbid mood disorder (MD)) and reference region (lDLPFC). Subject was used as a random effect. Abbreviations: SE = standard error; df = degrees of freedom.

. p ≤ .1, * p ≤ .05, ** p ≤ .01, *** p ≤ .001

**Table S6: Linear mixed effect model for gamma power including mood disorders**

|  | Estimate | SE | df | t-value | p |  |
| --- | --- | --- | --- | --- | --- | --- |
| Intercept | 1.18 | 0.58 | 43.08 | 2.02 | 0.0499 | * |
| DMPFC | -0.07 | 0.19 | 84 | -0.35 | 0.727 |  |
| lPPC | -0.05 | 0.19 | 84 | -0.26 | 0.7935 |  |
| MD | -0.51 | 0.30 | 83.12 | -1.67 | 0.0984 | . |
| Age | -0.00 | 0.03 | 40 | -0.17 | 0.8663 |  |
| Antipsychotics | 0.25 | 0.33 | 40 | 0.77 | 0.4449 |  |
| DMPFC x MD | -0.06 | 0.31 | 84 | -0.20 | 0.8461 |  |
| lPPC x MD | -0.41 | 0.31 | 84 | -1.32 | 0.1909 |  |

*Note*. Results of the linear mixed-effect model for gamma power. The intercept represents the reference group (no comorbid mood disorder (MD)) and reference region (lDLPFC). Subject was used as a random effect. Abbreviations: SE = standard error; df = degrees of freedom.

. p ≤ .1, * p ≤ .05, ** p ≤ .01, *** p ≤ .001

**Table S7: Correlation between theta power and BS/SOFAS**

|  | n | M | SD | lDLPFC | lPPC | DMPFC |
| --- | --- | --- | --- | --- | --- | --- |
| COPER | 44 | 11.8 | 9.6 | -0.26 | -0.11 | 0.01 |
| COGDIS | 44 | 8.4 | 7.7 | -0.18 | -0.17 | -0.11 |
| SOFAS | 37 | 59.7 | 10.2 | 0.17 | 0.08 | -0.00 |

*Note*. Result of the Spearman rank correlation between TMS-related theta power and basic symptom criteria item scores (COPER and COGDIS) as well as level of functioning (SOFAS).

**Table S8: Correlation between theta power and neurocognition**

|  | *n* | *M* | *SD* | lDLPFC | lPPC | DMPFC |
| --- | --- | --- | --- | --- | --- | --- |
| AVLT 1 | 43 | 46.9 | 27.5 | -0.13 | -0.19 | -0.11 |
| AVLT total | 43 | 50.6 | 33.3 | 0.04 | 0.09 | 0.03 |
| AVLT delayed | 43 | 44.7 | 32.5 | 0.08 | 0.15 | 0.11 |
| DSST | 42 | 10.8 | 5.8 | -0.13 | -0.04 | 0.15 |

*Note*. Result of the Spearman rank correlation between TMS-related theta power and neurocognitive test results. AVLT 1 represents the percentile reached in the first recall, total the percentile of all recalls and delayed the recall after 30 minutes of the Auditory Verbal Learning Test. DSST represents the standardized score in the Digit Symbol Substitution Test.

**Table S9: Correlation between gamma power and BS/SOFAS**

|  | *n* | *M* | *SD* | lDLPFC | lPPC | DMPFC |
| --- | --- | --- | --- | --- | --- | --- |
| COPER | 44 | 11.8 | 9.6 | 0.11 | 0.06 | -0.02 |
| COGDIS | 44 | 8.4 | 7.7 | 0.13 | 0.08 | -0.06 |
| SOFAS | 37 | 59.7 | 10.2 | -0.25 | -0.19 | -0.21 |

*Note*. Result of the Spearman rank correlation between TMS-related theta power and basic symptom criteria item scores (COPER and COGDIS) as well as level of functioning (SOFAS).

**Table S10: Correlation between gamma power and neurocognition**

|  | *n* | *M* | *SD* | lDLPFC | lPPC | DMPFC |
| --- | --- | --- | --- | --- | --- | --- |
| AVLT 1 | 43 | 46.9 | 27.5 | 0.01 | 0.15 | 0.08 |
| AVLT total | 43 | 50.6 | 33.3 | 0.13 | -0.09 | 0.03 |
| AVLT delayed | 43 | 44.7 | 32.5 | 0.10 | 0.00 | 0.04 |
| DSST | 42 | 10.8 | 5.8 | -0.03 | -0.05 | -0.05 |

*Note*. Result of the Spearman rank correlation between TMS-related gamma power and neurocognitive test results. AVLT 1 represents the percentile reached in the first recall, total the percentile of all recalls and delayed the recall after 30 minutes of the Auditory Verbal Learning Test. DSST represents the standardized score in the Digit Symbol Substitution Test.

Supplementary references

1. Zimmermann N, Koenig T, Riesen AS, Morishima Y (2025): Enhancing prefrontal modulation by phase-locking intermittent theta burst stimulation to a concurrent transcranial alternating current stimulation. *Imaging Neurosci* 3: imag_a_00415.

2. World Health Organization (2019): *International Statistical Classification of Diseases and Related Health Problems*, 11th ed.
